# How anaesthesiologists understand difficult airway guidelines—an interview study

**DOI:** 10.1080/03009734.2017.1406020

**Published:** 2018-01-04

**Authors:** Kati Knudsen, Ulrika Pöder, Ulrica Nilsson, Marieann Högman, Anders Larsson, Jan Larsson

**Affiliations:** a Department of Health and Caring Sciences, University of Gävle, Sweden; b Department of Public Health and Caring Sciences, Uppsala University, Sweden; c School of Health Sciences, Örebro University, Sweden; d Department of Medical Sciences, Uppsala University, Sweden; e Department of Surgical Sciences, Uppsala University, Sweden

**Keywords:** Airway guidelines, algorithms, qualitative study

## Abstract

**Background:**

In the practice of anaesthesia, clinical guidelines that aim to improve the safety of airway procedures have been developed. The aim of this study was to explore how anaesthesiologists understand or conceive of difficult airway management algorithms.

**Methods:**

A qualitative phenomenographic design was chosen to explore anaesthesiologists’ views on airway algorithms. Anaesthesiologists working in three hospitals were included. Individual face-to-face interviews were conducted.

**Results:**

Four different ways of understanding were identified, describing airway algorithms as: (A) a law-like rule for how to act in difficult airway situations; (B) a cognitive aid, an action plan for difficult airway situations; (C) a basis for developing flexible, personal action plans for the difficult airway; and (D) the experts’ consensus, a set of scientifically based guidelines for handling the difficult airway.

**Conclusions:**

The interviewed anaesthesiologists understood difficult airway management guidelines/algorithms very differently.

## Background

Managing endotracheal intubation in patients with a difficult airway during anaesthesia is a challenging task. Anaesthesiologists regularly handle situations in which the airway is compromised (1). In such situations, anaesthesiologists need both technical and non-technical skills (2), including competent communication and behaviour (3).

In order to improve safety in the management of the difficult airway and intubation in anaesthesia practice, clinical guidelines and algorithms have been developed (4). As an example, the Difficult Airway Society (DAS) recently presented an improved, simplified version of its algorithm (5). Even though the usefulness and evidence-base of guidelines have been questioned (6), algorithms seem to improve the ability of the anaesthesia staff to manage airway problems (7). There is, however, a wide variation in airway guidelines currently in use, and those guidelines have been reported to be difficult to implement (8).

One limitation of algorithms is that they are not applicable to all patients and situations; the patient’s individual anatomy and the clinical situation must also be considered (9). Moreover, human factors, such as personality, scientific beliefs, attitudes, and more, can influence anaesthesiologists in their understanding of algorithms (10).

Research in educational science has shown that behind differences in how people deal with something there are often differences in how people understand or conceive of that ‘something’ (11). To explain why anaesthesiologists use algorithms for the difficult airway in different ways, it is therefore of value to map the different conceptions of such algorithms among these professionals.

The aim of this study was therefore to explore how anaesthesiologists understand or conceive of difficult airway management algorithms.

## Methods

The study was approved by the Regional Ethics Review Board in Uppsala, Sweden (Dnr. 2014/491), on 14 January 2015. A phenomenographic design was chosen, and data were collected by interviews. Phenomenography describes variations in how people understand a specific phenomenon (11). The idea supporting this research approach is that in a group of people there is usually a limited number of qualitatively different ways of understanding or looking upon a certain phenomenon. By mapping these ways of understanding, the researcher can make explicit the thinking behind different ways of dealing with the phenomenon. The result of phenomenographic studies is usually presented as categories of description. In this study, we defined ‘algorithms for managing the difficult airway’ as the phenomenon.

### Participants and setting

Twenty anaesthesiologists from anaesthesiology departments in three hospitals (one university and two community hospitals) in Sweden participated in the study. The sample size was based on the experience of numerous phenomenographic studies that 15–20 interviews are enough to capture the variation in how the phenomenon is understood (12). Inclusion criteria were that the participants should be clinically active, specialized anaesthesiologists or trainees, with a minimum of 2 years of working experience in anaesthesia practice. We contacted the head of each department of anaesthesia by e-mail to request that a letter of invitation to the study be distributed. During workplace meetings at the three departments, the anaesthesiologists were informed about the study. The eligible volunteers were interviewed in a quiet room at their workplace after written informed consent had been obtained. All interviews were carried out by the first author of this study (K.K.) during a one-week interval at each workplace, between January and February 2015.

### Data collection

Individual, face-to-face interviews were conducted (13) with an interview guide that comprised three main, open-ended questions: 1) ‘Can you give an example of an event when you were involved in handling a predicted or an unpredicted difficult airway?’; 2) ‘What do you think about algorithms, also referred to as guidelines, protocols, or decision aids?’; and 3) ‘Can you describe a situation where you felt that you were successful in managing a difficult airway, and a situation where you were not successful?’ Probing questions were asked when necessary to keep the focus on airway algorithms. During the interviews, the terms ‘algorithms’, ‘guidelines’, ‘protocols’, and ‘decision support’ were used interchangeably, and no effort was made by the interviewer to distinguish between them. ‘Algorithms’ and ‘guidelines’ are the main terms used throughout this article, consistent with most publications in this field (4). Three pilot interviews with experienced anaesthesiologists were performed to test the interview guide, and minor adjustments were made. These interviews were not included in the study. The interviews lasted a median of 35 (range: 23–61) min. All interviews were audio-recorded and transcribed verbatim by a professional transcription company.

### Data analysis

The data analysis was carried out in five steps (13), as follows:

1. The print-outs were read and re-read several times by the first author (K.K.) to get an overall impression of the response to the interview.
2. On each interview print-out, text sections were marked where the anaesthesiologists described experiences of using algorithms in difficult airway situations or reflected on such experiences. These were considered as the parts of the interviews that were of value for the study.
3. For each interview, these marked text sections were condensed into a description of what the anaesthesiologist thought about algorithms. Each such summary represented a ‘preliminary way of understanding’.
4. Next, the resulting 20 ‘preliminary ways of understanding’ were compared and, based on similarities and differences, grouped into different categories by three of the authors (K.K., U.P., J.L.).
5. Each category was discussed to confirm its accuracy. When necessary, the interview texts were reviewed and the categories of description were revised.

During the analysis process, three of the authors discussed and reassessed the categories, reviewed the interview texts, and enhanced the rigour of the analysis (13); these three authors had experience in interviewing and in qualitative methods (K.K., U.P., J.L.). One author had experience specifically in the phenomenographic method (J.L.). All but one author had experience with difficult airways from work in anaesthesiology (K.K., U.N., M.H., A.L., J.L.).

### Availability of data and materials

The data analysed in the present study are available from the corresponding author on reasonable request.

## Results

Of the 20 participants, 5 were women and 15 were men, with a median age of 39 (range: 29–68) years. They had a median of 3.5 (range: 2–39) years of clinical experience as anaesthesiologists.

Four different ways of understanding were identified, describing airway algorithms as (A) a law-like rule, (B) a cognitive aid, (C) a basis for personal algorithms, and (D) the experts’ consensus. Several of the interviewees expressed more than one understanding, as seen in Table I. Each category is described below and exemplified with quotations from the interviews.

**Table I.** Ways of seeing algorithms for the difficult airway among 20 Swedish anaesthesiologists.

	Category (A)	Category (B)	Category (C)	Category (D)
Years of working experience	A law-like rule for how to act in difficult airway situations	A cognitive aid, an action plan for difficult airway situations	A basis for developing flexible, personal algorithms for the difficult airway	A set of scientifically based guidelines for handling the difficult airway, the experts’ consensus
2		X		
2		X		
2		X		
3	X	X		
3		X		
3		X		
3			X	
3			X	
3		X		X
3		X		X
4	X	X		X
4		X	X	X
5		X		
13		X	X	X
17	X	X	X	
18			X	X
20			X	X
20				X
26		X		
39			X	X

X = category presented.

### (A) An algorithm is a law-like rule for how to act in difficult airway situations

In this category, the algorithm is seen as a fixed, standardized norm, almost a law. When an incident has occurred, you must be able to defend your choice of action to avoid criticism. If things have gone seriously wrong, you may even be disciplined for not having followed the rule. If, for some reason, one has deviated from the algorithm, the reasons must be clearly declared.

‘A rigid algorithm will bind you, almost like the hand of the law.’

‘There are some sets of rules within our practice … e.g. never administer neuromuscular blockers before having tried face-mask ventilation. If you follow all these rules, I think you will end up in many unnecessarily dangerous situations. One should be able to go outside the rules without being questioned afterwards …’

### (B) An algorithm is a cognitive aid, an action plan for difficult airway situations

An airway algorithm can be understood as a succinct plan to follow in a difficult airway situation. It must be easy to understand, simple to memorize, and should include back-up plans. The algorithm can help the anaesthesiologist not to forget any important step in the procedure. However, a rigid algorithm with too many steps is difficult to memorize and may be a hindrance rather than a support in a critical situation.

An airway algorithm can help practitioners become familiar with the equipment used in managing difficult airways, during training, and in less stressful airway situations. It is important that anaesthesiology staffs are familiar with and have practised the algorithms used in their respective departments, to improve teamwork and prevent chaos.

‘Algorithms are there in the back of your mind … so that you have a plan to follow and know what to do next … but they’ve got to be simple and easy to follow, no more than 3 to 4 steps, to help avoid becoming blocked in critical situations … once you are in a difficult airway situation, you may not be able to think of every step in the algorithm …’

‘… during a crisis, you need something that you have mastered and practised in scenario training … when you get into a stressful and critical situation, you’d better have something simple …’

### (C) An algorithm is a basis for developing flexible, personal action plans for the difficult airway

The algorithm is to be used when creating an action plan based on experience, a personal, versatile plan, adaptable to the needs of the individual patient and the clinical situation. Looked upon in this way, algorithms are valuable tools that allow practitioners to hone their individual cognitive and behavioural skills, to promote excellence in managing difficult airways.

‘A plan of necessary actions that can be used to create your own personal algorithm, based on clinical experiences …’

‘An algorithm is a useful tool, but you need to tailor it for each individual patient … a useful support, like a crutch … but patients are also different; they have various conditions and anatomy, and one must take these factors into account …’

### (D) An algorithm is a scientifically based set of guidelines for handling the difficult airway, the experts’ consensus

In this category, algorithms are seen as a synthesis of scientific knowledge and experts’ consensus on how to act. Algorithms provide evidence-based guidance, ideas, and recommendations for how to act.

‘I feel comfortable with the fact that there exists a consensus among experienced colleagues who have described, in a structured way, how you can best handle difficult [airway] situations … there is, after all, a lot of competence behind these recommendations and guidelines …’

## Discussion

This study examined different ways of understanding algorithms for the difficult airway in a group of Swedish anaesthesiologists. We identified four different understandings, which we formulated as four categories of description.

As can be seen from Table I, about half of the interviewees expressed more than one understanding. This is in line with how the result of a phenomenographic study is seen by phenomenographic theorists: a structured description of the phenomenon under study, where the different categories represent different aspects of the phenomenon as experienced (14). In the best of cases, the anaesthesiologist is consciously aware of all aspects of airway algorithms and can deliberately focus on the most appropriate one, for instance in a teaching situation.

The first category was the algorithm seen as a law-like rule, a normative plan that specifies what an anaesthesiologist must do to avoid criticism after an incident. One problem with this way of seeing algorithms is that it could dilute individual responsibility and have a negative impact on clinical learning. The importance of using cognitive aids, such as algorithms, during critical situations has been pointed out (4,7). However, if such aids are used as strict law-like rules, clinical learning could be hampered, and the anaesthesiologist may need more time to learn the more advanced, nuanced ways of managing the airway. A possible advantage of strict norms is that they could prevent patient harm.

The second category was that the algorithm was understood as a cognitive aid in difficult airway situations, with a potential to lead the anaesthesiologist through all steps in the management of difficult airway situations, which could instil confidence in the stressed anaesthesiologist. However, in time-pressured situations, algorithms can be difficult to adhere to, and a plan that is not well designed can divert focus from the actual situation by occupying too much of the anaesthesiologist’s cognitive capacity (15).

In the third category, algorithms were seen as a basis for developing flexible, personal plans for action, using one’s own individual experience and skill. Thus, simulation training could be one method to incorporate individual clinical experience and skills into a personal algorithm, to fit multiple specific situations.

Some anaesthesiologists, seeing algorithms as *law-like rules*, *cognitive aids*, or *bases for a personal action plan*, did not express any considerations for scientific evidence, as in category D, *the experts’ consensus*. This may well be an example of a characteristic of phenomenographic studies: the most robust result of such studies is a structured way of describing the phenomenon, whereas which aspects are covered by the individual interviews is more of a coincidence (16). The *experts’ consensus* way of understanding algorithms may actually be quite common (and perhaps seen as self-evident by some anaesthesiologists), since it has been shown that a cognitive aid cannot be implemented without an evidence base. However, one problem with experts’ consensus is that experts’ opinions may differ; therefore, it can be difficult to choose which guideline to use. On the other hand, several anaesthesia societies (4,5) recommend guidelines based on the best available research and consensus of experts, with the intention of minimizing variation in practice and improving patient safety.

A recurring statement in all interviews was that the ability to use algorithms requires training and clinical experience. Such training can, for instance, be carried out in a simulator setting. Although simulation scenarios cannot emulate the multifaceted and unpredicted clinical reality, simulator training has been found to importantly improve procedural skills and performance in critical situations (7), including situation awareness, decision-making, teamwork, and leadership (3).

A high-risk profession which has approached, in a systematic way, the kind of professional challenges discussed here is aviation. Most airline pilots are examined twice a year in terms of both technical and non-technical skills to ensure that they can operate the aircraft safely. Moreover, they undergo simulator training regularly, and using checklists is an integrated component of their work (17). In anaesthesia practice, in contrast, regular training in the use of airway algorithms is not mandatory in many countries, notwithstanding the beneficial effect this could have on patient safety. However, when algorithms are applied too rigidly, they may negatively affect flexibility in specific situations (6,18).

Adherence to evidence-based guidelines has been related to both the clinical organization and the patient’s situation (19), but also to the knowledge and beliefs of the individual anaesthesiologists (10). Managing a difficult airway is a complex task, especially outside the anaesthesia department, because the assistance may be inadequate. In such situations, an algorithm could be a useful tool for the whole team by reminding them of the next step of the procedure.

The four ways, A to D, of understanding algorithms can be seen as the parallel stages of development from novice to expert first described by Dreyfus and Dreyfus (20). In clinical practice, beginners with no previous experience make decisions based on strict rules; in contrast, experts with extensive clinical experience may see situations differently, and identify and handle problems more intuitively. The highest level of expertise is the master who bases most decisions on intuition, but who, in tutoring situations, can link a clinical event with scientific theory (21), thus encouraging trainees to make that link. In our study, the masters can be found in the small group of anaesthesiologists who combine versatile intuition (C) with explicit scientific knowledge (D). Larsson et al. (22) found that support from more senior anaesthesiologists was important for trainees when they faced difficult situations at work. This implies that although an algorithm is a guide in difficult airway situations, guidance from more experienced colleagues should be available.

In general, the participants of the present study considered the algorithms for difficult airway management valuable tools for decision-making. However, they also mentioned that algorithms are not always easy to follow. For instance, because algorithms are constructed to be used as a general tool, they do not consider a patient’s individual anatomical characteristics (e.g. a tonsillar tumour) or a specific situation. In this regard, a strict adherence to the algorithm is not always applicable in clinical situations and might not improve patient safety.

Kapur et al. argued (17) that the health-care sector has much to learn from the aviation industry. We partly agree with this statement, but we should consider that we are taking care of patients with individual characteristics and comorbidities, whereas airplanes are very standardized devices that hopefully have been carefully checked before use. In this sense, in our view, the application of conclusions from aviation to health care has to be done with great caution.

Our results were consistent with findings by Borges et al. (23) who showed that compliance with difficult airway guidelines depended on human factors, including the professionals’ experience regarding making decisions in critical situations. Furthermore, Borges et al. (23) concluded that the consideration of following or deviating from guidelines is influenced by the anaesthesiologists’ beliefs about the consequences of their actions. This could explain why algorithms are not used as much as is recommended (8). In addition, our interviews indicated that the algorithms should be based on expert opinion and easy to follow. The new clinical guideline recently proposed by DAS fulfils many of these requirements (5). It is based on a simple principle: maintaining oxygenation is more important than initiating ventilation; moreover, it is a consensus document written by leading world experts, and it comprises very few steps. However, to improve compliance with difficult airway guidelines, uncomplicated assessment tools and teaching materials have to be developed. Improving procedural skills in critical airway situations requires hands-on training, in addition to the support of more experienced colleagues (24).

This study had the limitations common to most qualitative studies, namely that the sample size is small and that the findings may not be fully generalizable. However, in our study we included both men and women of various ages, with different clinical experience levels, and from different hospitals, to capture as many different understandings of the algorithms as possible. Therefore, we believe that the results from this study will be valid for hospital systems with levels of education and training in anaesthesia similar to those common in hospital systems in Sweden.

In this study, we have mapped the different ways anaesthesiologists understand airway algorithms, exploring through in-depth interviews their thinking about such guidelines. Follow-up observational studies in theatre would be valuable, to link the anaesthetists’ different ways of understanding algorithms with actual handling of airway problems in clinical situations.

In conclusion, we found that anaesthesiologists understood airway algorithms in four different ways: (A) as a law-like rule for how to act in difficult airway situations; (B) as a cognitive aid, an action plan for difficult airway situations; (C) as a basis for developing flexible, personal algorithms for the difficult airway; and (D) as a scientifically based set of guidelines for handling the difficult airway, the experts’ consensus. These different ways of understanding airway algorithms could explain variations among anaesthesiologists with regard to their attitudes towards such guidelines.
